# Bile lakes in patients with biliary atresia who presented with jaundice-free native liver survival indicating the risk of subsequent liver transplantation due to various factors

**DOI:** 10.1007/s00383-024-05786-x

**Published:** 2024-07-17

**Authors:** Yousuke Gohda, Hiroo Uchida, Wataru Sumida, Chiyoe Shirota, Takahisa Tainaka, Satoshi Makita, Miwa Satomi, Akihiro Yasui, Daiki Kato, Takuya Maeda, Hiroki Ishii, Kazuki Ota, Yaohui Guo, Jiahui Liu, Akinari Hinoki

**Affiliations:** 1 Department of Pediatric Surgery, Nagoya University Graduate School of Medicine, 65 Tsurumai-cho, Showa-ku, Nagoya, 466-8550 Japan; 2 Department of Rare/Intractable Cancer Analysis Research, Nagoya University Graduate School of Medicine, 65 Tsurumai-cho, Showa-ku, Nagoya, 466-8550 Japan

**Keywords:** Biliary atresia, Liver transplantation, Bile lake, Intrahepatic cystic lesion, Risk score

## Abstract

**Purpose:**

The prognostic factors of subsequent liver transplantation (LT) in patients with biliary atresia (BA) who presented with jaundice-free native liver survival were investigated.

**Methods:**

This study retrospectively reviewed patients who underwent portoenterostomy (PE) for BA. Patients with jaundice-free native liver survival at 1 year postoperatively were divided into the autologous liver survivor and liver transplant recipient groups. Peri- and postoperative data were compared between the two groups.

**Results:**

Among 97 patients with BA, 29 who received LT within 1 year after PE were excluded from the analysis. Further, 48 patients currently living with native liver and 20 who received LT after 1 year postoperatively were compared. Bile lake (BL) was the strongest risk factor of LT. The risk score was $2.38*BLscore+0.00466*TBA$, and the area under the receiver operating characteristic curve was 0.83. Patients with BL and those without significantly differed in terms of the native liver survival rate. Patients with BL who presented with not only cholangitis but also gastrointestinal hemorrhage and hepatopulmonary syndrome received LT.

**Conclusion:**

BL can cause different pathologies. Moreover, it is an evident risk factor of subsequent LT in patients with BA who are living with native liver at 1 year after PE.

## Background

Biliary atresia (BA) is a progressive disease that presents with cholestasis, leading to liver fibrosis in early infancy. Portoenterostomy (PE) is the standard first-line treatment for BA. Approximately 50–60% of patients with BA have experienced jaundice clearance. Finally, 14.9–51% of patients with BA were alive with native liver after PE [1–5]. Other patients with BA require liver transplantation (LT) due to non-improvement in jaundice, progressive portal hypertension, uncontrolled cholangitis, hepatopulmonary syndrome, and gastrointestinal hemorrhage. LT is an effective salvage therapy for these patients. However, it has a disadvantage. That is, it requires a donor liver and lifelong immunosuppression. Therefore, research has focused on identifying the long-term outcomes of PE to predict prognosis and appropriately assess the need for LT. Previous studies investigated various factors associated with LT. Results showed that only age at KP is the common prognostic factor of LT [1–5]. These reports analyzed the prognosis of patients with BA at the time of PE. In contrast, the risk factor of future LT in stable patients living with native liver if they have survived for some time after PE is unclear. Based on the Kaplan–Meier curves, patients with native liver survival (NLS) had a high rate of LT during the immediate postoperative period, particularly within 1 year after surgery [2, 6]. Therefore, the risk of LT differs from the patients with BA who live with jaundice-free native liver at 1 year postoperatively and those who required LT before. It is important to be knowledgeable on whether patients with postoperative BA who presented with jaundice-free native liver survival until 1 year of age will be stable or will require LT [7]. Hence, the prognostic factor of future LT at 1 year after PE in patients with BA who were living with native liver and not scheduled for LT was investigated. Moreover, a risk score for future LT was developed using the date up to 1 year postoperatively.

## Materials and methods

### Patients

This study retrospectively reviewed patients who underwent PE for BA between January 2008 and February 2021. Patients with jaundice-free native liver who were not scheduled for LT at 1 year after PE were included in this analysis. The patients were divided in the autologous liver survivor and liver transplant recipient groups. The perioperative and 1-year postoperative data of the two groups were compared. The data included demographic characteristics of the patients including cirrhosis diagnosed by pathological findings at the first surgery, pre- and postoperative blood test values (avoiding the period of cholangitis), history of gastrointestinal bleeding, and number of cholangitis, the presence of bile lake (BL) at 1 year postoperatively, timing of BL occurrence. Diagnostic criteria of BL were an intrahepatic cystic lesions detected by abdominal ultrasonography or computed tomography scan. BL may include irregular dilation of the intrahepatic bile ducts. Quantitative variables were expressed as median and interquartile range (IQR). Qualitative variables were expressed as number and percentages. To identify prognostic factors, univariate analyses were first conducted using the Mann–Whitney *U* test for continuous variables and the Fisher’s exact test for categorical variables. Patients with missing values were excluded from comparison. After identifying the prognostic parameters by univariate analysis, multivariate logistic regression analysis (best-subset selection method) was performed to identify the risk factors independently affecting prognosis and to develop the risk score formula. The number of independent variables to be input in one multivariate analysis was estimated according to the number of sample size. The formula of risk score was developed using best-subset selection method, combining the independent risk factors and other variables. Coefficients of parameters were derived from the estimate value in the logistic regression analysis. The model that maximized the receiver operating characteristic curve (AUC) and minimized the number of variables was adopted. The predictive performance of the adopted models was evaluated by assessing its discrimination and calibration. Discrimination was measured using AUC, sensitivity, specificity and accuracy. Calibration was measured using the Hosmer and Lemeshow goodness of fit test. For the prognostic factors, NLS was analyzed using the Kaplan–Meier curve and the log-rank test. *P*-values of < 0.05 were considered statistically significant. Statistical analyses were performed using EZR (Saitama Medical Center, Jichi Medical University, Saitama, Japan). This study was conducted based on the 1964 Declaration of Helsinki and its later amendments. Further, it was approved by the institutional review board (approval number: 2023–0403). Anonymous clinical data were used in the analysis. Patients were not required to provide a written informed consent. However, the opt-out method was applied to obtain consent for this study.

### Protocol after PE

The patients received oral prednisolone at a dose of 4 mg/kg/day starting on postoperative day 5. The dose was tapered to half of the dose every 5 days. Patients who had a total bilirubin level of ≤ 2.0 mg/dL and who had been receiving prednisolone at a dose of ≤ 1 mg/kg/day were discharged. The patients visited the outpatient clinics, and the prednisolone dose was reduced, and then discontinued. The patients visited the outpatient clinics within a short period immediately after discharge and finally every 3 months. Blood test and abdominal ultrasonography were performed during each outpatient clinic visit.

## Results

In total, 97 patients underwent PE for BA during the study period. Further, 29 patients who received LT within 1 year after PE were excluded from the study, and 68 patients were included in this analysis. Forty-eight patients currently live with native liver (NLS group), and 20 patients live with liver transplantation (LT group). The mean age at LT was 2.7 (IQR: 1.6–4.6) years. Table 1 shows the demographic characteristics of the patients. The two groups did not significantly differ in terms of patient’s demographics. The mean ages at PE were not significantly late at 58.5 days in the NLS group and 55.0 in the LT group.

**Table 1:** Demographic characteristics of the patients

	NLS group (*n* = 48)	LT group (*n* = 20)	*P* value
Sex (male/female), *n*	21/27	6/14	0.42
Type of BA (I/III)	4/44	0/20	0.31
Age at PE, days (IQR)	58.5 (40.8–67.5)	55 (52.0–77.3)	0.83
Cirrhosis, *n* (%)	5 (10%)	2 (10%)	1.00
Follow-up period, years (IQR)	10.0 (6.3–13.2)	8.6 (6.6–10.1)	0.39

*IQR* interquartile range

Table 2 shows the parameters at 1 year postoperatively. The two groups significantly differed in terms of total bilirubin (TB), direct bilirubin (DB), γ-GTP, cholinesterase (ChE), total bile acid (TBA), and type IV collagen levels and the presence of BL.

**Table 2:** Univariate analyses of data at 1 year postoperatively

	*n*	NLS group (*n* = 48)	*n*	LT group (*n* = 20)	*P* value
AST, U/L (IQR)	48	64.0 (44.0–98.5)	20	101.0 (77.5–122.0)	0.09
ALT, U/L (IQR)	48	46.5 (27.5–79.5)	20	54.0 (48.5–81.8)	0.21
TB, mg/dL (IQR)	48	0.50 (0.40–0.70)	20	0.80 (0.65–1.00)	0.00*
DB, mg/dL (IQR)	48	0.10 (0.10–0.10)	20	0.20 (0.10–0.43)	0.00*
γ-GTP, U/L (IQR)	48	100.0 (34.8–174.0)	20	166.5 (110.5–231.0)	0.02*
ChE, U/L (IQR)	36	292.5 (245.8–366.3)	18	228.0 (193.8–301.5)	0.02*
TBA, μmol/L (IQR)	27	61.8 (22.9–134.3)	17	141.8 (55.2–296.5)	0.04*
M2BpGi (IQR)	23	2.40 (0.87–3.47)	14	2.78 (2.23–4.67)	0.06
Hyaluronic acid, ng/mL (IQR)	29	77.0 (31.0–148.0)	15	93.0 (65.5–138.5)	0.28
TypeIV collagen, ng/mL (IQR)	13	266.0 (201.0–346.0)	6	629.0 (432.8–743.5)	0.01*
History of bleeding, n (%)	48	2 (4%)	20	4 (20%)	0.06
Cholangitis, n (IQR)	48	1 (0.0–2.0)	20	1 (0–2.3)	0.97
Presence of BL, n (%)	48	7 (15%)	20	12 (60%)	0.00*
Timing of BL, day (IQR)	48	161.5 (101.3–212.5)	20	228.0 (188.5–262.0)	**0.08**

*IQR* interquartile range

Bold and asterisk (*) indicate a significant difference (*P* < 0.05)

Among those identified prognosis factors and the age at PE, multiple logistic regression analysis was performed. Type IV collagen level was excluded due to insufficient sample size. The number of variables to be input in one analysis was set to be 2 according to the sample size. Whichever factor was mixed, only BL commonly had a significant impact on prognosis (Table 3). BL was the most influential factor.

**Table 3:** Multivariate analyses with BL and other parameters

Combination of variables	Variables	Odds ratio (95% CI)	*P* value
BL and Age at PE	BL	8.80 (2.63–29.40)	0.00*
	Age	1.00 (0.97–1.03)	0.98
BL and TB	BL	7.54 (1.93–29.40)	0.00*
	TB	1.38 (0.33–5.72)	0.66
BL and DB	BL	7.92 (2.21–28.40)	0.00*
	DB	1.66 (0.18–15.50)	0.66
BL and γ-GTP	BL	8.71 (2.48–30.60)	0.00*
	γ-GTP	1.00 (1.00–1.00)	0.97
BL and ChE	BL	7.88 (1.81–34.3)	0.01*
	ChE	1.00 (0.99–1.01)	0.51
BL and TBA	BL	10.80 (2.40–48.8)	0.00*
	TBA	1.00 (1.00–1.01)	0.09

*CI* confidence interval

Bold and asterisk (*) indicate a significant difference (*P* < 0.05)

Therefore, BL was used in combination with the other variables to develop the risk score of future LT. Several models were developed based on the result of multiple logistic regression analysis and AUC. However, if the coefficients did not match the prognostic impact of a parameter, the model was not adopted. For example, a higher γ-GTP level can lead to a worse prognosis. However, if the coefficient was negative and a higher γ-GTP level indicated a decreased risk, the model was not adopted. Finally, two model was adopted.

Model 1: $$2.07*BLscore+0.505*DB$$ (*P* = 0.00).

Model 2: $$2.38*BLscore+0.00466*TBA$$ (*P* = 0.00).

(BL score: if bile lake was present, the score = 1; if not, the score = 0).

**Fig. 1:**
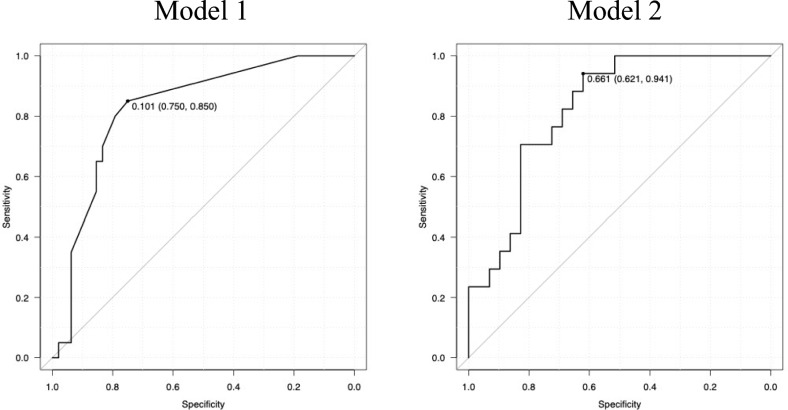
Area under the receiver operating characteristic curve of the risk score for predicting future LT

The AUCs of BL alone and models 1 and 2 were 0.73 (95% CI: 0.61–0.85, *P* = 0.00), 0.83 (95% CI: 0.73–0.93), and 0.83 (95% CI: 0.71–0.94), respectively (Fig. 1). Adding other variables did not improve AUC. The two model was compared, and they did not significantly differ (*P* = 0.28). If cutoff point was set to 0.101 in Model 1, discrimination was 0.85 (95% CI: 0.62–0.97) for sensitivity, 0.75 (95% CI: 0.60–0.86) for specificity, 0.78 (95% CI: 0.66–0.87) for accuracy. If cutoff point was set to 0.661 in Model 2, discrimination was 0.88 (95% CI: 0.64–0.99) for sensitivity, 0.67 (95% CI: 0.46–0.84) for specificity, 0.75 (95% CI: 0.60–0.87) for accuracy. Calibration measured using the Hosmer and Lemeshow goodness of fit test were 0.03 and 0.55 for *P* value in Models 1 and 2, respectively. Based on the result of discrimination and calibration, we selected Model 2.

Model 1: $2.07*BLscore+0.505*DB$ AUC: 0.83 (95% CI: 0.73–0.93).

Model 2: $2.38*BLscore+0.00466*TBA$ AUC: 0.83 (95% CI: 0.71–0.94).

**Fig. 2:**
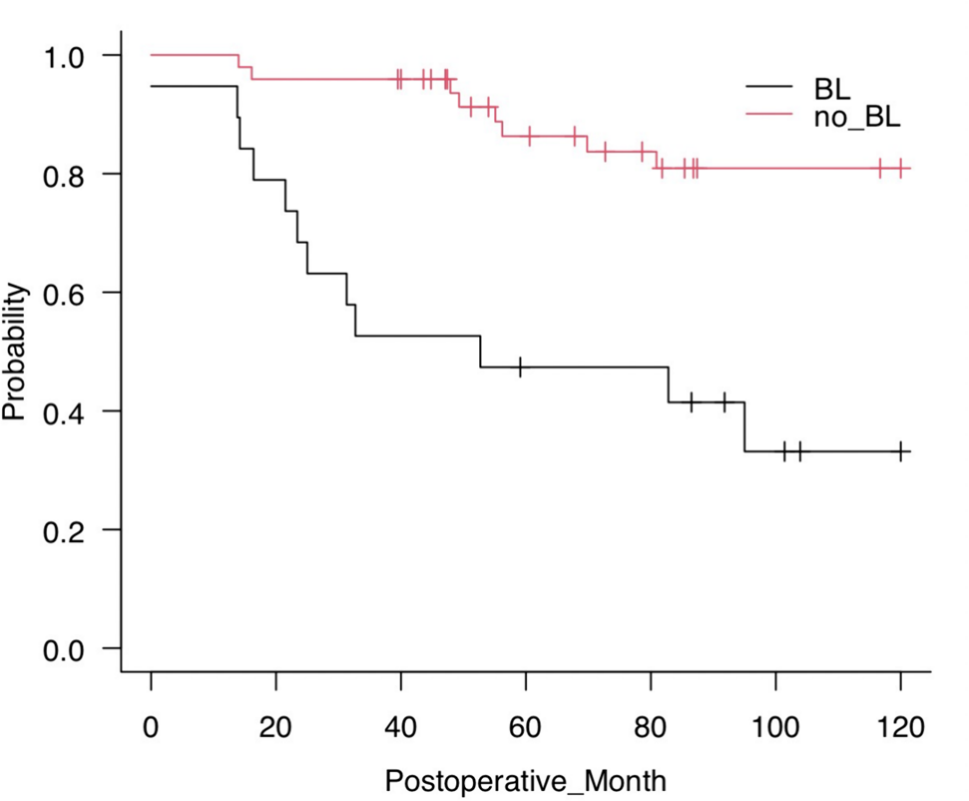
The native liver survival rate significantly differed between patient who had BL at 1 year after KP and those who did not (*P* = 0.00)

Figure 2 shows the Kaplan–Meier curves of NLS among patients living with native liver at 1 year after PE who presented with BL and those who did not. Patients with BL had a high risk of LT in the long-term (*P* = 0.00).

Table 4 shows the causes of LT between patients who had BL at 1 year after PE and at LT and those who did not. The presence of BL was not significantly related to the cause of LT.

**Table 4:** Causes of liver transplantation

Causes of LT	With BL/without BL at POY 1 (*n* = 12/8)	*P* value	With BL/without BL at LT (*n* = 13/7)	*P* value
Recurrence of jaundice, *n*	1/3	0.26	1/3	0.10
Uncontrollable cholangitis, *n*	5/2	0.64	6/1	0.33
Repeated gastrointestinal bleeding, *n*	3/1	0.62	2/2	0.59
Hepatopulmonary syndrome, *n*	1/2	0.54	2/1	1.00
Growth retardation associated with impaired liver function, *n*	2/0	0.50	2/0	0.52

*POY* postoperative one year

## Discussion

This study evaluated the different risk factors of future LT for patients with BA living with jaundice-free native liver at 1 year after PE. The patients with BA have lifelong risk for LT and require lifelong follow-up. They have various life events ahead and it is important to know the risk of LT for decision on appropriate follow-up intervals, scheduling of detailed examinations and, for women, the timing of pregnancy. Furthermore, prognostic model is needed to accurately and objectively predict prognosis. These factors included the demographic characteristics of the patients, blood test values, presence of BL and cholangitis, and history of bleeding. Among these parameters, the presence of BL was the strongest risk factor of future LT.

BL is an intrahepatic cystic lesion that occurs after PE in 6.4%–26% of patients with BA [8]. The prognosis of patients with BA who presented with BL had been investigated, and BL was considered a risk factor of cholangitis, which leads to LT [8–12]. In particular, compared with a simple cyst, cyst with a shape type such as multiple or bead-like patterns is associated with a worse prognosis [12–14]. In our study, at year 1 postoperatively, the presence of BL was the most influential risk factor for subsequent LT. Other parameters were found to have an impact on prognosis in the univariate analysis. However, none of the factors affected prognosis in the multivariate analysis with BL. By contrast, some parameters were useful in predicting prognosis. The AUC can improve if DB and TBA are added as prognostic parameters, in contrast to other parameters. Therefore, the two models were developed with DB and TBA. The predictive ability of the two models was comparable. However, calibration of Model 1 was not good. Hence, Model 2 was selected.

The LT group did not have a higher number of cholangitis until 1 year postoperatively than the NLS group. Furthermore, we found no significant difference in the number of cholangitis in comparison between the patients with and without BL (1 vs. 1, *P* = 0.252). The rate of cholangitis is generally high in the early postoperative period, and the development of BL takes time [10, 15]. Therefore, BL and LT were not related to the number of cholangitis up to 1 year after PE. Another research has reported similar results. That is, BL did not increase the number of cholangitis in the short term [9]. The research also reported that BL increases the number of cholangitis in the long term. Patients living with native liver at 1 year postoperatively can have long-term NLS. Hence, so comparison in only those patients in our study might have identified BL as a risk factor for subsequent LT. Further, based on the Kaplan–Meier curves, patients with BL had a significantly poor prognosis with consideration of the passage of time. In relation to its natural course, BL rarely improves. Thus, if BL occurs, it can increase the risk of LT during the rest of the patient’s life. Although this was a 10-year study, patient prognosis may still differ within in a longer period of time.

The causes of subsequent LT vary. The most common cause was uncontrollable cholangitis in patients with BL. However, uncontrollable cholangitis was the cause of LT in less than half of all cases. According to previous studies reporting an association between cholangitis and BL, BL may be a cause of uncontrollable cholangitis. However, there was no significant difference in the incidence of uncontrollable cholangitis between patients with and without BL. BL with cholangitis was actively treated via percutaneous transhepatic biliary drainage or internal intestinal drainage in our institution [8, 14, 16, 17]. As a result, two patients in the LT group and four in the NLS group had a successfully controlled cholangitis after drainage. Recurrent cholangitis with BL did not always lead to LT. Our study revealed that in the presence of BL, not only cholangitis but also hepatopulmonary syndrome, gastrointestinal hemorrhage, and growth retardation with impaired liver function have resulted in LT. Thus, BL can be associated with progressive fibrosis of the liver and poor liver function.

Age at surgery, which is the most common prognostic factor, had no significant impact on future LT in patients with BA living with native liver at 1 year after PE. This result might have been caused by different demographic characteristics in the current and previous research. Previous studies investigating age at surgery have analyzed all types of patients, including those who have never been cleared of jaundice [1, 2, 4]. However, this study only analyzed jaundice-free patients with native liver survival at 1 year postoperatively. Patients who were not successfully cleared of jaundice often require early LT. Therefore, none of these patients was included in our study. Due to this difference in cohort, age at surgery could not be considered a prognostic factor at this time point.

Relationship between the liver tissue findings at the first surgery and the occurrence of bile lake was confirmed in this investigation. In total, 27 patients finally experienced the occurrence of bile lake until now. Of these, four patients pathologically diagnosed with cirrhosis. The degree of fibrosis and inflammation varied and ranged from mild to severe. No relationship seemed to exist between the severity and the occurrence of bile lake. Cholestasis was not described in detail and could not be considered. We confirmed this from the pathology at PE, and we will investigate it in more detail.

This study had several limitations. First, it had a retrospective, historical-control, single-center design. Hence, multicenter prospective studies should be performed for a more accurate analysis There were only two parameters included in the multivariate analysis due to the small sample size. BA is a rare disease. Therefore, a multicenter analysis should be performed to collect a large sample size. The risk of BL was investigated. However, various characteristics, such as the number, form, and size, of were not compared. The follow-up period was at least 3 years. Nevertheless, patients with BA occasionally require LT 3 years after PE. Hence, further follow-up is required to consider long-term prognosis. Although, we checked the usefulness of the risk score model by discrimination and calibration, cross validation was required for assessing the accuracy of the model in more detail. However, the number of the cases in this study was insufficient for the analyses. Therefore, we will test the usefulness of the model either by accumulating more cases or using data from other institutions.

## Conclusion

The presence of BL at 1 year after PE was a strong risk factor of future LT in patients with BA living with native liver. Further, using this factor, a risk score for predicting future LT was developed.

## Data Availability

No datasets were generated or analysed during the current study.
